# Age-related macular degeneration

**Published:** 2014

**Authors:** Wanjiku Mathenge

**Affiliations:** Africa Regional Medical Advisor: Fred Hollows Foundation, Kigali, Rwanda. ciku@email.com

**Figure:**
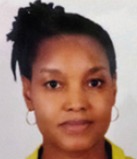
Wanjiku Mathenge

## What is AMD?

Age-related macular degeneration (AMD) is a disease of the retina that usually develops in people aged 60 years and older. It affects about 8.7% of the world's population and is the leading cause of blindness among people aged 50 and older in industrialised countries.^1^

AMD affects the macula. When it becomes advanced, it destroys the central vision we use to look straight ahead. This is necessary for recognising faces, reading books or using mobile phone screens, watching television, sewing, preparing food, driving, safely navigating stairs and performing other daily tasks we take for granted. If the macula is damaged, the picture is there but the fine points are not clear.

Fortunately, the peripheral vision remains intact. This means that some patients with AMD will retain some independence, and eye workers should reassure them that peripheral vision will not be lost, even if no treatment is possible.

## Is it increasing in low- and middle-income countries?

A recent review of the global prevalence of AMD shows that the number of people with AMD in 2020 is projected to be 196 million, which will increase to 288 million in 2040.^1^ Studies of AMD in low- and middle-income countries have shown that, in contrast to what was originally thought, AMD is not rare in Asian and African populations but is instead a significant contributor to blindness. Table 1 shows the prevalence from some recent studies involving different ethnic groups.

## Classification

AMD can be classified as either early-stage or late-stage. In the early stage, AMD is characterised by atrophy or hypertrophy of the retinal pigment epithelium (RPE) underlying the central macula, as well as drusen deposition. (Drusen are deposits of extracellular material lying between the basement membrane of the RPE and the inner collagen layer of Bruch's membrane beneath the RPE.)

**Figure:**
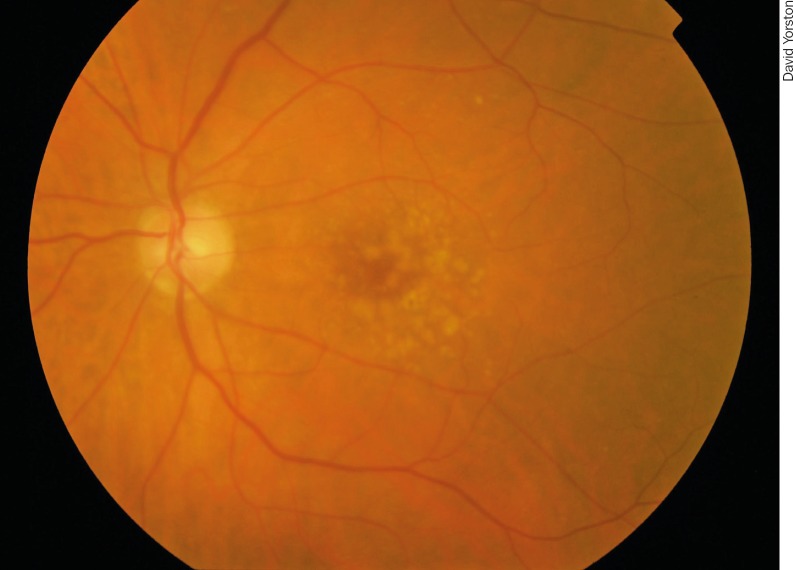
Early AMD. There are irregular pale dots at the macula, which are called drusen. They are caused by a build-up of waste products from photoreceptor metabolism. Although drusen are associated with AMD, most patients with drusen will not develop severe AMD

The early stages of AMD may progress to either atrophic (‘dry’) or exudative (‘wet’) AMD. It is these advanced stages that are associated with vision impairment.

In **atrophic AMD** there is atrophy of the central macula, with gradual destruction of the RPE and the photoreceptors.

In **exudative AMD**, abnormal choroidal vessels/capillaries (pathologic choroidal neovascular membranes) develop under the macula, leak fluid and blood, and, ultimately, cause a central fibrous sub-retinal scar, with destruction of the photoreceptors and retinal pigment epithelium. Approximately 10–20% of patients with atrophic AMD can progress to the exudative form.

## Risk factors

Susceptibility to AMD is influenced by increasing age, smoking and family history. Smoking is the most consistent risk factor associated with advanced AMD in the majority of the prevalence studies.

Several genetic variants that influence susceptibility to AMD have recently been identified. People who have one or more of these genetic variations are at particularly high risk of developing AMD if they also smoke.

Three types of nutritional factors have been investigated for their potential protection against eye ageing: antioxidants (mainly zinc and vitamins C and E), the carotenoids lutein and xeanthine and omega-3 polyunsaturated fatty acids. Unfortunately, the results of supplementation have been disappointing, as large doses must be taken daily for the remainder of the patient's life and the benefit, if any, is small.

**Table 1.** Prevalence of AMD in recent studies

Author; Study	Dates and country	Number of subjects (N); age	Prevalence of late AMD (%)
**La; Korean National Health and Nutrition Survey**	2008–2011, Korea	N = 14,352; ≥ 50 years	0.6
**Mathenge; Nakuru Posterior Segment Eye Study**	2007–2008, Kenya	N = 3,304; ≥ 50 years	1.2
**Kawasaki; Funagata Study**	2000–2002, Japan	N = 1,037; ≥ 55 years	0.8
**Krishnan; INDEYE**	2005–2007, India	N = 4,266; ≥ 60 years	1.2
**Korb; European cohort: Gutenberg Health Study**	2007–2012, Germany	N = 4,340; 35–74 years	0.2

## How it presents

AMD occurs in both eyes, but it is often asymmetric. In the early stage, patients are often without symptoms, or sometimes they notice mild symptoms such as minimally blurred central visual acuity, reduced contrast, changes in the way colour is seen, and mild metamorphopsia (distortion of visual images).

Patients who develop atrophic AMD may notice a scotoma (blind spot), which slowly enlarges over months or years before becoming stable. This particularly affects reading.

Patients with exudative AMD typically describe painless progressive blurring of their central visual acuity, which usually occurs quite rapidly, over a few weeks. Patients also report relative or absolute central scotomas, metamorphopsia and difficulty with reading.

**Using the Amsler grid for self-testing**

The Amsler grid can be given to patients with early AMD (and any other patients over 60 years of age) for self-testing.

The Amsler grid can help the person spot macular defects early and tell their eye care worker about any increase in the distortion they see (which indicates increasing damage). Those reporting distortion should visit an ophthalmologist for further tests. If someone has a normal test, they should continue testing at regular intervals.

If an Amsler grid is unavailable, people can test themselves for distortion by looking at a straight edge or a right angle, such as a door frame or window, with one eye at a time. If they notice any distortion, they should contact their nearest eye care or health care worker and request referral to an ophthalmologist.

Early detection of wet AMD is critical because treatment, when indicated, is most successful when performed before damage occurs.

**Figure:**
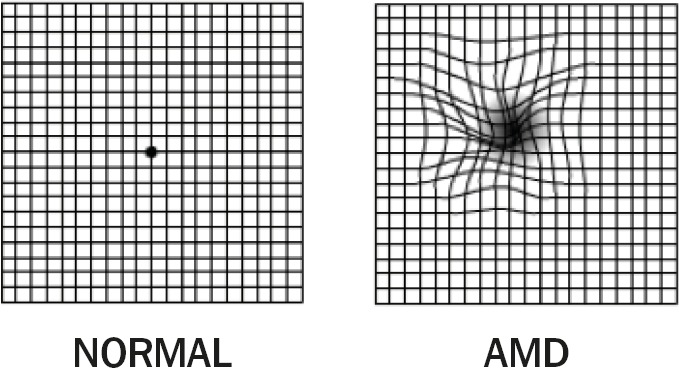

The natural history of exudative AMD or occasionally atrophic AMD results in a stable central scotoma in which the visual acuity falls below the reading level and the legal driving level. With exudative AMD, the visual outcome can be much worse. However, peripheral vision is usually retained.

## At the district level

With the advent of effective therapy for the neovascular form of AMD, early diagnosis and treatment is recommended and there is increased emphasis on patient self-screening for the early symptoms of disease. The most important preventive measure is to stop smoking.

If a patient over the age of 60 years presents with any symptoms of AMD, visual acuity should be tested and recorded. At the primary level, all patients with reduced vision should be referred to the eye clinic for further assessment. At the district hospital, the macula can be examined for the presence of drusen (see image on page 49) or pigment changes at the macula. Drusen can be seen as pale yellow deposits. If these are present then an Amsler grid test may be carried out.

The Amsler grid is a test that can be used in clinics to screen people over 60 years of age. It can also be taught to patients with early AMD for self-testing. An Amsler grid consists of straight lines, with a reference dot in the centre. Each eye is tested separately. The patient is advised to hold the chart at the normal reading distance and to cover one eye. While focusing only on the central dot, the patient describes whether she or he sees any distortions in the grid pattern.

Someone with macular degeneration may see some or all of the following:

- Straight lines that appear wavy or bent
- Boxes that differ in size or shape from the others
- Lines that are missing, blurry or discoloured
- Dark areas at the centre of the grid.

Patients with an abnormal Amsler grid test should be referred to an ophthalmologist.

## Investigations

The ophthalmologist's initial examination of patients with signs and symptoms of AMD should include visual acuity testing and a thorough stereo examination of the macula using a biomicroscopy lens (60–90D). This is often followed by imaging studies such as:

- **Stereo colour photography of the fundus:** for establishing, documenting, and tracking the exact size of the lesion.
- **Fundus fluorescein angiography (FFA):** the gold standard for diagnosing choroidal new vessels (CNV) due to AMD. Facilities performing FFA must have an emergency care plan and a protocol to minimise the risk and to manage any complications.
- **Optical coherence tomography (OCT):** excellent at detecting increased retinal thickness due to leakage from the abnormal vessels. This is a simpler, faster and safer investigation than FFA, but OCT machines are still very expensive.

## Management

Until recently, ophthalmologists used laser destruction of abnormal vessels/capillaries as the primary treatment for exudative AMD.^2^ These procedures included thermal laser photocoagulation and later the inclusion of intravascular photosensitisers such as verteporfin used in photodynamic therapy. However, at best these treatments slowed progression of the condition. They were not expected to lead to any improvement in vision.

The treatment of exudative AMD changed dramatically with the advent of vascular endothelial growth factor (VEGF) inhibitors (see articles on pages 44–48). Pharmaceutical drugs have been developed to block or neutralise VEGF in patients with AMD. These include pegaptanib (Macugen), ranibizumab (Lucentis), bevacizumab (Avastin), and aflibercept (Eylea). These are given as intravitreal injections and several doses are needed. They have been shown to stabilise vision in most patients with exudative AMD, and many patients will experience a significant improvement in visual acuity.^3^

There are no effective treatments for atrophic AMD at present. Patients should be reassured that progression is usually slow and they are likely to retain their independence even if reading vision is compromised. Other useful interventions may include smoking cessation, rehabilitation and low vision aids. The latter two are important in improving patients' quality of life, and health workers should make patients aware of these options and how to access them.
